# Clinical Determinants of Serum Uric Acid Levels in Patients with Obesity and Hypertension

**DOI:** 10.3390/jcm15145438

**Published:** 2026-07-11

**Authors:** Beata Moczulska, Karolina Osowiecka, Anna Bryczkowska, Natalia Jaje-Rykowska, Leszek Gromadziński, Marta Majewska

**Affiliations:** 1 Department of Cardiology and Internal Medicine, School of Medicine, University of Warmia and Mazury in Olsztyn, Warszawska 30, 10-082 Olsztyn, Poland; anna.bryczkowska@uwm.edu.pl (A.B.); natalia.jaje@uwm.edu.pl (N.J.-R.); leszek.gromadzinski@uwm.edu.pl (L.G.); 2 Internal Medicine Clinical Ward, University Clinical Hospital, Warszawska 30, 10-082 Olsztyn, Poland; 3 Department of Psychology and Sociology of Health and Public Health, School of Public Health, University of Warmia and Mazury in Olsztyn, Warszawska 30, 10-082 Olsztyn, Poland; karolina.osowiecka@uwm.edu.pl; 4 Department of Human Physiology and Pathophysiology, School of Medicine, University of Warmia and Mazury in Olsztyn, Warszawska 30, 10-082 Olsztyn, Poland; marta.majewska@uwm.edu.pl

**Keywords:** body mass index, cardiometabolic risk, hypertension, hyperuricemia, metabolic syndrome, obesity

## Abstract

**Background:** Hyperuricemia is increasingly recognized as an important component of metabolic dysfunction and cardiometabolic risk, particularly in individuals with obesity and hypertension. However, the relative contribution of obesity-related metabolic disturbances and blood pressure parameters to serum uric acid levels remains incompletely understood. The aim of this study was to identify independent clinical and metabolic determinants of serum uric acid levels and to determine whether obesity or hypertension is more strongly associated with elevated serum uric acid levels. **Methods**: This retrospective observational study evaluated the metabolic determinants of serum uric acid levels in 370 hospitalized adults stratified according to obesity and hypertension status. Serum uric acid concentrations were measured using an enzymatic spectrophotometric method and compared across four clinical groups, and univariate and multivariable analyses were performed to identify independent determinants of serum uric acid levels. Allopurinol use was recorded and included in the multivariable analysis, whereas the effects of individual antihypertensive drug classes were not evaluated. **Results**: Hyperuricemia was identified in 16.8% of the cohort, with the highest prevalence observed among patients with coexisting obesity and hypertension (22%). Serum uric acid levels were higher in men than in women (median: 6.4 vs. 5.7 mg/dL; *p* < 0.001). In the multivariable analysis, higher expected serum uric acid levels were independently associated with higher BMI, higher ALT activity, eGFR ≤ 90 mL/min/1.73 m^2^, and male sex, whereas older age and not receiving allopurinol were independently associated with lower expected serum uric acid levels. Each 1 kg/m^2^ increase in BMI was associated with an approximately 0.48% higher expected serum uric acid level (95% CI: 0.19–0.78%; *p* = 0.001). Blood pressure parameters were not significant predictors. **Conclusions**: These findings suggest that excess adiposity and associated metabolic disturbances may play a more prominent role than hypertension alone in the development of hyperuricemia. Targeting obesity and metabolic risk factors may therefore represent an important strategy for reducing hyperuricemia and improving cardiometabolic health.

## 1. Introduction

Hyperuricemia is increasingly recognized as an important metabolic and cardiovascular risk factor associated with obesity, hypertension, and chronic kidney disease. The global burden of hyperuricemia has increased substantially in recent decades. A large systematic review and modelling analysis including nearly 7.5 million adults estimated that, between 2000 and 2023, the global prevalence of hyperuricemia increased from approximately 6% to 11% in women and from approximately 12% to 18% in men. Over the same period, the estimated number of affected individuals increased from 126 million to 305 million women and from 226 million to 500 million men [1]. In the United States, data from the National Health and Nutrition Examination Survey (NHANES) indicated that hyperuricemia affects approximately 20% of both women and men [2]. These epidemiological trends highlight the growing clinical and public health importance of hyperuricemia.

Uric acid is the final product of purine metabolism, and its accumulation in the bloodstream occurs when its production increases or renal excretion decreases, leading to hyperuricemia [3]. Hyperuricemia has been linked to the development of several metabolic and cardiovascular disorders, including gout, hypertension, diabetes mellitus, atrial fibrillation, ischemic heart disease, heart failure, and chronic kidney disease [4]. The underlying mechanisms involve oxidative stress, endothelial dysfunction and inflammation [5]. Experimental and clinical studies have further suggested that elevated serum uric acid levels may contribute to the development of hypertension through several interrelated mechanisms [6]. Excess uric acid reduces nitric oxide (NO) bioavailability, activates pro-inflammatory pathways, and enhances oxidative stress, leading to endothelial dysfunction and impaired vasodilation [3]. At the same time, hyperuricemia promotes vascular smooth muscle cell proliferation and activates the renin–angiotensin–aldosterone system, leading to vascular remodeling. Together with impaired vasodilation, these changes increase vascular resistance and contribute to further elevations in blood pressure [7].

The relationship among obesity, hypertension, and serum uric acid levels has been recognized for many years. Elevated uric acid levels may contribute to hypertension development, while chronic hypertension may impair renal function and reduce uric acid excretion [8,9,10]. Importantly, the association between obesity and hyperuricemia may reflect not only excess body mass but also obesity-related metabolic dysfunction. An excess of metabolically active adipose tissue is associated with increased xanthine oxidase activity, a key enzyme involved in uric acid production. Chronic inflammation and oxidative stress may further enhance xanthine oxidase activity, thereby promoting uric acid production and contributing to elevated serum uric acid levels [11,12]. In addition, insulin resistance associated with obesity may impair renal urate excretion, further contributing to hyperuricemia [13]. These mechanisms may be particularly relevant in central and visceral adiposity, which is more closely associated with metabolic dysfunction than BMI alone.

Therefore, early recognition of hyperuricemia may be important for preventing cardiovascular and metabolic complications. Obesity and hypertension are highly prevalent, frequently coexist, and are both associated with elevated serum uric acid levels and increased cardiometabolic risk. However, because excess adiposity, blood pressure elevation, renal dysfunction, and metabolic abnormalities are closely interrelated, the independent contribution of obesity and hypertension to serum uric acid levels is difficult to disentangle. Despite well-established associations between hyperuricemia, obesity, and hypertension, it remains unclear which clinical and metabolic factors are most strongly and independently associated with serum uric acid levels in patients affected by obesity and/or hypertension. Although numerous studies have examined the associations of hyperuricemia with obesity and hypertension separately, direct comparisons of their relative contributions to serum uric acid levels, particularly in patients stratified according to the coexistence of both conditions and with simultaneous consideration of relevant clinical and metabolic factors, remain limited. Therefore, the aim of the present study was to identify independent clinical and metabolic determinants of serum uric acid levels in patients stratified according to obesity and hypertension status and to determine whether obesity or hypertension is more strongly associated with elevated serum uric acid levels. Understanding these relationships may provide further insight into the metabolic mechanisms underlying hyperuricemia and help identify patients who may benefit from closer assessment of cardiometabolic risk.

## 2. Materials and Methods

### 2.1. Study Population

This retrospective observational study included patients hospitalized at the Department of Cardiology and Internal Diseases of the University Clinical Hospital in Olsztyn, Poland, between 2017 and 2024. The study population comprised patients admitted primarily for evaluation and management of inadequately controlled hypertension or for comprehensive internal medicine assessment before planned bariatric surgery. The study period was predefined, and all consecutive patients hospitalized during this period were assessed for eligibility. Patients with acute or recent infections, fever, active malignancy, chronic liver disease, chronic lung disease, heart failure, or previously diagnosed chronic kidney disease were excluded to reduce potential confounding by conditions that could influence serum uric acid levels or metabolic and inflammatory parameters. The final study cohort comprised 370 eligible patients. No formal a priori sample size calculation was performed because of the retrospective observational design; therefore, the final sample size was determined by the number of eligible patients available during the predefined study period.

### 2.2. Clinical Assessment

Based on the presence or absence of hypertension and obesity, the study population was divided into four groups: patients without obesity and hypertension (Group 1: Ob−/Ht−), patients without obesity but with hypertension (Group 2: Ob−/Ht+), patients with obesity but without hypertension (Group 3: Ob+/Ht−), and patients with both obesity and hypertension (Group 4: Ob+/Ht+). Hypertension was defined on the basis of a previous diagnosis of hypertension documented in the patients’ medical history. Systolic and diastolic blood pressure values were obtained from routine hospital medical records. Blood pressure measurements were performed as part of standard clinical care in accordance with routine hospital practice. The measurement procedures were consistent with standard recommendations for office blood pressure assessment [14]. Obesity was defined as a body mass index (BMI) ≥ 30 kg/m^2^ according to the World Health Organization criteria [15].

### 2.3. Laboratory Measurements

Venous blood samples were collected on the day of hospital admission after an overnight fast and before the administration of the patients’ usual morning medications, according to standard hospital laboratory procedures. Laboratory analyses were performed using routine validated methods in a certified diagnostic laboratory and included measurements of C-reactive protein (CRP), fasting glucose, liver enzymes, thyroid-stimulating hormone (TSH), serum creatinine, serum uric acid, and a lipid profile. Serum uric acid concentrations were determined using an enzymatic spectrophotometric method. Clinical symptoms potentially related to hyperuricemia were not systematically assessed; therefore, hyperuricemia was defined solely by serum uric acid concentrations. Hyperuricemia was diagnosed when the serum uric acid concentration exceeded 7.0 mg/dL in men and 6.0 mg/dL in women [1]. The study was conducted in accordance with the Declaration of Helsinki and was approved by the Bioethics Committee of the School of Medicine of the University of Warmia and Mazury in Olsztyn on 22 June 2017 (number 28/2017).

### 2.4. Statistical Analysis

The normality of continuous variables was assessed using the Shapiro–Wilk test. As all continuous variables were found to have non-normal distributions, they were summarized as medians with interquartile ranges (IQRs), whereas categorical variables were presented as frequencies and percentages. For comparisons of non-normally distributed continuous variables among the four study groups, the Kruskal–Wallis test was applied. Following a significant result, post hoc pairwise comparisons were conducted using Dunn’s test to control for multiple testing. Differences among the four study groups were evaluated using the chi-square test for categorical variables. The frequency of elevated serum uric acid levels across the study groups stratified by sex was compared using the chi-square test. Differences in serum uric acid levels according to selected dichotomous variables were assessed using the Mann–Whitney U test. Associations between serum uric acid level and continuous variables were evaluated using Spearman’s rank correlation coefficient. To assess the independent associations between serum uric acid level and selected explanatory variables, a generalized linear model (GLM) with a logarithmic link function was fitted. The final model was selected based on the Akaike information criterion (AIC), choosing the specification that provided the best fit to the data. Statistical significance was set at *p*-value < 0.05. Statistical analyses were performed using Statistica software version 13 (https://www.statsoft.pl; StatSoft, Poland).

## 3. Results

### 3.1. Clinical and Metabolic Characteristics According to Obesity and Hypertension Status

The final study cohort comprised 370 patients, including 183 women (49.5%) and 187 men (50.5%). The median age of the overall study population was 48 years (IQR: 38–61). The highest proportion of smokers was observed in the group of patients with both obesity and hypertension (Ob+/Ht+). This group also included the largest number of patients with diabetes mellitus, depression, and hypothyroidism. Hyperuricemia was identified in 62 patients (16.8%) across the entire study population, with the highest prevalence observed in the Ob+/Ht+ group (22%). Allopurinol use differed significantly across the four study groups: 1 patient in the Ob−/Ht− group, 3 patients in the Ob−/Ht+ group, 6 patients in the Ob+/Ht− group, and 41 patients in the Ob+/Ht+ group (*p* < 0.001). Thus, the highest prevalence of allopurinol use was observed in the group with coexisting obesity and hypertension. Notably, despite the highest prevalence of allopurinol use, this group also exhibited the highest serum uric acid levels (Table 1).

Significant differences in the age distribution were observed between the study groups (*p* < 0.001). The oldest patients were in the Ob−/Ht+ group, with a median age of 66 years, whereas the youngest patients were in the Ob+/Ht− group, with a median age of 37 years. Post hoc analysis revealed statistically significant differences among all groups except the comparison between Ob−/Ht− and Ob+/Ht+. The proportion of women was significantly higher in the Ob+/Ht− group than in the other groups (*p* < 0.001). The median BMI values were significantly higher in the Ob+/Ht− and Ob+/Ht+ groups than in the Ob−/Ht− and Ob−/Ht+ groups (*p* < 0.001). Moreover, diabetes mellitus and hyperuricemia occurred significantly more frequently in patients with both obesity and hypertension than in those in the other study groups (*p* < 0.05) (Table 1).

Patients with both obesity and hypertension exhibited significantly higher total cholesterol (TC) levels than patients without obesity and with hypertension, and they also showed significantly lower high-density lipoprotein (HDL) cholesterol concentrations. In addition, obese patients without hypertension had lower HDL cholesterol levels than non-obese patients with hypertension. Regardless of the hypertension status, obese patients had significantly higher triglyceride (TG) levels than non-obese patients. Similar patterns were observed for CRP and TSH, with significantly higher values recorded in obese individuals than in non-obese subjects. Patients with hypertension had significantly higher fasting glucose levels than those without hypertension, irrespective of their BMI. The same trend was observed for serum creatinine concentrations (Table 2).

**Figure 1:**
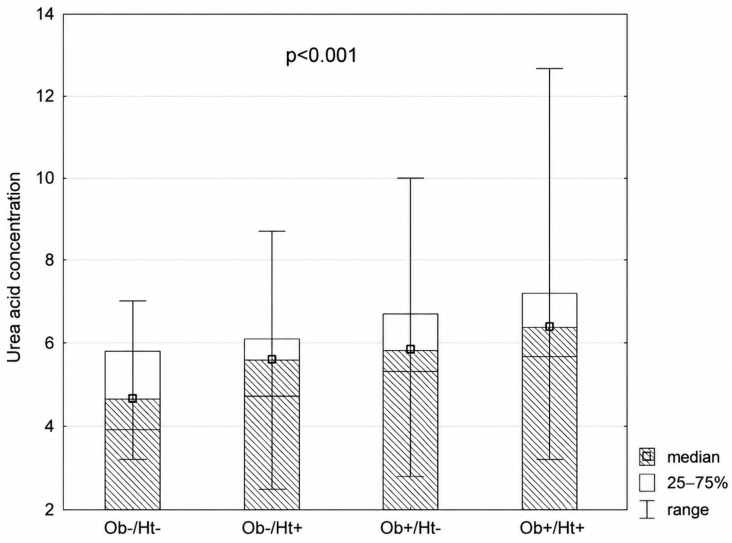
Comparison of serum uric acid levels among patients without obesity and hypertension (Ob−/Ht−), without obesity and with hypertension (Ob−/Ht+), with obesity and without hypertension (Ob+/Ht−), and with both obesity and hypertension (Ob+/Ht+). Values are expressed as medians with interquartile ranges and total ranges (*p* < 0.001).

Post-hoc analysis revealed significant differences in serum uric acid levels between the following subgroups: Ob−/Ht− vs. Ob+/Ht− (*p* < 0.001), Ob−/Ht− vs. Ob+/Ht+ (*p* < 0.001), and Ob−/Ht+ vs. Ob+/Ht+ (*p* < 0.001). Patients with obesity, irrespective of the presence of hypertension, exhibited significantly higher serum uric acid levels than non-obese, normotensive individuals. Among the patients with hypertension, significantly higher serum uric acid levels were observed in obese subjects than in non-obese (*p* < 0.001) (Figure 1).

### 3.2. Univariate Analysis

The median serum uric acid level was significantly higher in men than in women, reaching 6.35 mg/dL (IQR: 5.70–7.30) in men and 5.70 mg/dL (IQR: 4.80–6.30) in women (*p* < 0.001) (Figure S1). The highest prevalence of hyperuricemia, defined as serum uric acid >7 mg/dL in men and >6 mg/dL in women, was observed in men in the Ob+/Ht− group (52%). In this subgroup, elevated serum uric acid levels were significantly more frequent among men than among women (52% vs. 27%, *p* = 0.03). No significant sex-related differences in the distribution of hyperuricemia were observed among the remaining study groups (Figure S2). Notably, none of the men in the Ob−/Ht− group presented with hyperuricemia. Serum uric acid levels were significantly higher in patients with an eGFR ≤ 90 mL/min/1.73 m^2^ than in those with eGFR > 90 mL/min/1.73 m^2^, with median values of 6.4 mg/dL and 5.9 mg/dL, respectively (*p* = 0.04). Serum uric acid levels were significantly higher in patients receiving allopurinol than in those not receiving allopurinol (median: 7.4 vs. 5.9 mg/dL, respectively; *p* < 0.001). No significant differences in serum uric acid levels were observed with respect to smoking status, presence of diabetes mellitus, hypothyroidism, or depression (*p* > 0.05) (Table S1).

In the univariate analysis, significant positive linear correlations were observed between serum uric acid level and the following variables: BMI, TG, non-high-density lipoprotein cholesterol (non-HDL-C), CRP, ALT, AST, TSH, fasting glucose, systolic blood pressure on admission, and diastolic blood pressure on admission (all *p* < 0.05) (Figure S3). Significant negative linear correlations were identified between serum uric acid level and age, as well as HDL levels (*p* < 0.05). No significant correlations were found between the serum uric acid level and TC, LDL, or serum potassium levels (*p* > 0.05) (Table S2).

### 3.3. Multivariable Analysis

In the multivariable generalized linear model with a log link function, higher expected serum uric acid levels were independently associated with higher BMI, higher ALT activity, eGFR ≤ 90 mL/min/1.73 m^2^, male sex, and allopurinol use, whereas older age was independently associated with lower expected serum uric acid levels (Table 3). Each 1 kg/m^2^ increase in BMI was associated with an approximately 0.48% higher expected serum uric acid level (95% CI: 0.19–0.78%; *p* = 0.001). Each 1 U/L increase in ALT activity was associated with an approximately 0.14% higher expected serum uric acid level (95% CI: 0.00–0.28%; *p* = 0.043). Patients with an eGFR ≤ 90 mL/min/1.73 m^2^ had an approximately 5.2% higher expected serum uric acid level than those with an eGFR > 90 mL/min/1.73 m^2^ (95% CI: 0.3–10.4%; *p* = 0.038). Men had an approximately 6.1% higher expected serum uric acid level than women (95% CI: 2.3–10.1%; *p* = 0.002). Patients not receiving allopurinol had an approximately 7.2% lower expected serum uric acid level than those receiving allopurinol (95% CI: −10.0% to −4.2%; *p* < 0.001). Finally, each additional year of age was associated with an approximately 0.25% lower expected serum uric acid level (95% CI: −0.46% to −0.03%; *p* = 0.026) (Table 3).

## 4. Discussion

In the present study, we investigated the relationship between obesity, hypertension, and serum uric acid levels in a cohort of hospitalized patients. The main finding of our analysis was that obesity showed a stronger association with elevated serum uric acid levels than hypertension. Although the highest uric acid levels were observed in patients with both obesity and hypertension, multivariable analysis demonstrated that body mass index remained an independent determinant of uric acid level, whereas blood pressure parameters did not.

The relationship between hyperuricemia and hypertension has been well documented in epidemiological studies. Previous reports indicate that hyperuricemia occurs in approximately 20–40% of patients with hypertension, whereas hypertension is present in 25–50% of individuals with gout. Epidemiological data suggest that the risk of developing hypertension may increase even at serum uric acid levels around 5.5 mg/dL, which is below the conventional threshold used to define hyperuricemia [16]. Interestingly, studies on adolescents with primary hypertension have reported hyperuricemia in up to 90% of patients, suggesting that elevated uric acid levels may precede the development of hypertension early in life [11]. These findings highlight the strong clinical overlap between these conditions and support the concept that elevated serum uric acid may represent an important cardiometabolic risk factor.

Consistent with these observations, our findings demonstrated that patients with coexisting obesity and hypertension had the highest serum uric acid levels. These observations indicate that metabolic disturbances associated with excess adiposity may contribute substantially to elevated serum uric acid levels. Several mechanisms may explain the association between obesity and elevated serum uric acid levels. Increased adiposity promotes insulin resistance and reduces renal uric acid excretion, whereas metabolic and inflammatory changes associated with obesity may further increase uric acid production.

In line with these mechanisms, our findings suggest that excess adiposity, rather than elevated blood pressure, may be the primary metabolic driver of hyperuricemia in this patient population. Although the highest uric acid levels were observed in patients with coexisting obesity and hypertension, multivariable analysis demonstrated that body mass index remained an independent determinant of uric acid level, whereas blood pressure parameters did not. These findings emphasize the potential role of adiposity-related metabolic disturbances, such as insulin resistance and impaired renal urate handling, in the development of hyperuricemia. Taken together, these observations support the concept that metabolic disturbances associated with obesity may represent an upstream driver of hyperuricemia, whereas hypertension may emerge as a downstream consequence within the cardiometabolic continuum.

In our cohort, hyperuricemia was diagnosed in 62 patients (16.8%), and the highest prevalence was observed in individuals with both obesity and hypertension (22%). The lower prevalence observed in our study may be explained by the relatively young age of our population, with a median age of 48 years. Hyperuricemia has been associated with several adverse health outcomes, including chronic kidney disease, cardiovascular disease, metabolic syndrome, and diabetes mellitus [17,18]. Simultaneously, these conditions may contribute to elevated uric acid levels, creating a complex bidirectional relationship between hyperuricemia and metabolic disorders.

Our results further support the concept that metabolic factors related to excess adiposity are important determinants of serum uric acid levels. Although patients with both obesity and hypertension exhibited the highest uric acid levels, the multivariable analysis showed that BMI remained an independent determinant of uric acid level. This observation supports the hypothesis that metabolic disturbances associated with excess adiposity, including insulin resistance, increased purine turnover, and impaired renal urate excretion, contribute substantially to the development of hyperuricemia.

The association between obesity and elevated serum uric acid levels may be driven not only by excess body mass but also by the metabolic dysfunction associated with central and visceral adiposity. Visceral adipose tissue is metabolically active and releases adipokines, pro-inflammatory cytokines, and other mediators that promote chronic low-grade inflammation, oxidative stress, and insulin resistance [19,20]. These metabolic disturbances may contribute to hyperuricemia through two complementary mechanisms: increased uric acid production and reduced renal urate excretion. Increased metabolic activity and purine turnover associated with excess adipose tissue may enhance uric acid production [19], whereas insulin resistance and compensatory hyperinsulinemia may reduce renal urate excretion by promoting tubular reabsorption of sodium and urate [20]. This relationship may also be bidirectional, as elevated uric acid levels may further aggravate oxidative stress and insulin resistance [21].

The close relationship between uric acid metabolism and the metabolic abnormalities associated with visceral adiposity is also reflected in the frequent coexistence of hypertriglyceridemia and reduced HDL cholesterol levels [20]. Importantly, imaging-based studies have demonstrated that serum uric acid is associated with visceral and hepatic fat accumulation and that these associations may persist after adjustment for conventional anthropometric measures [22]. In the present study, patients with obesity exhibited higher triglyceride and CRP levels and lower HDL cholesterol concentrations than non-obese patients. These findings support the possibility that the association between BMI and serum uric acid levels observed in our cohort reflects, at least in part, broader obesity-related metabolic dysfunction rather than excess body mass alone. However, because waist circumference and imaging-based measures of adiposity were not available, the independent contribution of central and visceral adiposity could not be directly assessed.

Serum uric acid levels also differ between men and women, which is reflected in the sex-specific diagnostic thresholds. In our study, hyperuricemia was more frequently observed in men, which is consistent with previous reports [23]. Hormonal factors may partially explain these differences. Estrogens appear to exert a protective effect by increasing renal uric acid excretion, whereas testosterone may promote urate reabsorption. In addition, differences in body fat distribution, with greater visceral adiposity in men and more peripheral fat distribution in women, may contribute to sex-related variations in uric acid metabolism. Su et al. reported a higher prevalence of obesity and hyperuricemia among women; however, their study population was older, suggesting that hormonal changes associated with ageing may modify this relationship [23]. Furthermore, declining estrogen levels after menopause may contribute to increased uric acid concentrations in older women.

Age also appears to influence the epidemiology of hyperuricemia. Li et al. reported that the prevalence of hyperuricemia was nearly 48% in individuals aged 65 years and older [24]. In contrast, Zhang et al. demonstrated an association between obesity and hyperuricemia even in children and adolescents with a mean age of 11 years [25]. Interestingly, the prevalence observed in that pediatric population (approximately 22%) was comparable to that reported in the adult populations. These findings suggest that the relationship between obesity and hyperuricemia develops early in life and persists throughout adulthood.

Our findings are also consistent with those of previous studies indicating that obesity may be closely linked with hyperuricemia and may contribute to pathways associated with the development of hypertension. Hong et al. demonstrated that elevated uric acid levels partially mediate the relationship between obesity and incident hypertension [26]. The experimental and clinical observations further support this concept. Mazzali et al. suggested that the early management of hyperuricemia may delay the development of essential hypertension [27]. Moreover, studies in adolescents have shown that hyperuricemia is present in up to 90% of patients with newly diagnosed primary hypertension, highlighting the potential role of uric acid in the early stages of blood pressure elevation [28]. In contrast, Liu et al. found that hyperuricemia occurred in nearly 39% of patients with hypertension, particularly among women older than 65 years, and reported that antihypertensive treatment was associated with lower uric acid levels [29]. These findings highlight the complex interactions between metabolic factors, renal function, and blood pressure regulation.

Hyperuricemia has also been associated with increased levels of inflammatory markers, including C-reactive protein, interleukin-6, and neutrophil counts, similar to obesity [30]. Therefore, some authors have suggested that hyperuricemia may represent an additional component of metabolic syndrome [31]. Moreover, several studies have demonstrated that elevated serum uric acid levels are independently associated with increased all-cause mortality, further emphasizing the clinical relevance of this condition [32,33]. The management of hyperuricemia remains challenging and extends beyond pharmacological treatment. Current recommendations emphasize a comprehensive approach including dietary modification, weight reduction, physical activity, and optimal management of coexisting metabolic disorders. In patients with high cardiovascular risk, early identification and treatment of hyperuricemia may play an important role in cardiovascular risk reduction [34,35,36]. These findings suggest that evaluation of metabolic status, particularly obesity, may be important when assessing hyperuricemia in patients with hypertension.

A strength of this study is the relatively large cohort of hospitalized patients and the use of multivariable analysis to evaluate independent determinants of serum uric acid levels. The generalizability of these findings should, however, be considered in light of the study setting. The present cohort consisted exclusively of patients hospitalized at a single center, who may differ from community-based populations in terms of comorbidity burden, metabolic risk profile, medication use, and healthcare utilization. Although a similar general pattern of association between excess adiposity and serum uric acid levels may also be observed in the general population, the magnitude of these associations cannot be assumed to be the same. Therefore, the present findings should be extrapolated to community-based populations with caution and require confirmation in population-based and multicenter cohorts. Future studies incorporating waist circumference, waist-to-height ratio, and imaging-based measures of visceral adiposity may provide further insight into whether the association between obesity and hyperuricemia is driven primarily by overall adiposity or by the metabolic dysfunction associated with central and visceral fat accumulation. Longitudinal, prospective studies are also needed to clarify the temporal and potentially causal relationships among excess adiposity, hyperuricemia, and hypertension and should include comprehensive assessment of metabolic dysfunction, renal function, medication use, and long-term cardiovascular outcomes. A better understanding of these relationships may help identify patients who could benefit from more comprehensive diagnostic evaluation and targeted preventive or therapeutic interventions. Because serum uric acid measurement is widely available and inexpensive, its potential value as a biomarker for cardiometabolic risk stratification warrants further investigation.

## 5. Conclusions

Obesity and hypertension are recognized risk factors for hyperuricemia; however, our findings indicate that excess adiposity may play a more prominent role than hypertension alone in determining elevated serum uric acid levels. These results highlight obesity as an important metabolic determinant of hyperuricemia. Effective management of hyperuricemia may benefit from comprehensive lifestyle interventions, particularly weight reduction and optimal control of metabolic risk factors.

## 6. Limitations

This study had several limitations. First, its retrospective design may have introduced potential selection bias. Second, the study population was derived from a single centre, which may limit the generalizability of the findings. Third, adiposity was assessed using BMI, as waist circumference was not systematically measured. Consequently, indices of central adiposity, such as waist circumference and waist-to-height ratio, could not be evaluated. Because BMI does not distinguish between visceral and peripheral adiposity, it may not fully capture obesity-related metabolic dysfunction relevant to uric acid metabolism. Fourth, the effects of individual classes of antihypertensive medications, including diuretics, on serum uric acid levels were not evaluated, potentially introducing residual confounding. In addition, dietary factors, alcohol consumption, direct measures of insulin resistance, and markers of increased uric acid production were not available in the retrospective dataset and could not be evaluated. These unmeasured factors may have influenced serum uric acid levels and contributed to residual confounding. In addition, because specific markers of subclinical renal injury, such as albuminuria or the urinary albumin-to-creatinine ratio, were not available in the retrospective dataset, early renal involvement could not be definitively excluded. Despite these limitations, the study included a relatively large cohort of patients and provided clinically relevant insights into the relationship between obesity, hypertension, and serum uric acid levels.

## Figures and Tables

**Table 1:** Baseline demographic, clinical, and anthropometric characteristics of the study population stratified according to obesity and hypertension status (Group 1 (Ob−/Ht−): patients without obesity and without hypertension; Group 2 (Ob−/Ht+): patients without obesity and with hypertension; Group 3 (Ob+/Ht−): patients with obesity and without hypertension; Group 4 (Ob+/Ht+): patients with both obesity and hypertension). Continuous variables are presented as medians with interquartile ranges (IQRs), and categorical variables as counts and percentages. *p*-values refer to comparisons between the four groups. BMI, body mass index.

Parameter *n* (%) or Median (IQR)		Total (*n* = 370)	Gr 1 (*n* = 34)	Gr 2 (*n* = 61)	Gr 3 (*n* = 77)	Gr 4 (*n* = 198)	*p*-Value
Age (years)		48 (38–61)	49.5 (37–66)	66 (55–70)	37 (29–45)	49 (39–59)	<0.001
Sex, *n* (%)	Female	183 (49.5)	14 (41)	25 (41)	55 (71)	89 (45)	<0.001
	Male	187 (50.5)	20 (59)	36 (59)	22 (29)	109 (55)	<0.001
BMI		38 (29.6–45.0)	24 (22.2–26.3)	26.2 (24.6–27.7)	42 (36.3–47.5)	41.9 (35.6–47.3)	<0.001
Smoking, *n* (%)		107 (28.9)	6 (18)	13 (21)	27 (35)	61 (31)	0.134
Diabetes, *n* (%)		88 (23.8)	6 (18)	14 (23)	9 (12)	59 (30)	0.012
Hypothyroidism, *n* (%)		51 (13.8)	3 (9)	9 (15)	10 (13)	29 (15)	<0.001
Depression, *n* (%)		33 (8.9)	1 (3)	9 (15)	6 (8)	17 (9)	0.241
Hyperuricemia, *n* (%)		62 (16.8)	1 (3)	3 (5)	14 (18)	44 (22)	0.002
Allopurinol, *n* (%)		51 (13.8)	1 (3)	3 (5)	6 (8)	41 (21)	<0.001

**Table 2:** Biochemical and clinical parameters of the study population, stratified by group assignment (Group 1: Ob−/Ht−; Group 2: Ob−/Ht+; Group 3: Ob+/Ht−; Group 4: Ob+/Ht+). Continuous variables are presented as medians with interquartile ranges (IQRs). Categorical variables are presented as counts and percentages. *p*-values refer to comparisons between the four study groups. Abbreviations: TC, total cholesterol; LDL, low-density lipoprotein cholesterol; HDL, high-density lipoprotein cholesterol; TG, triglycerides; non-HDL-C, non-high-density lipoprotein cholesterol (calculated as total cholesterol minus HDL cholesterol); eGFR, estimated glomerular filtration rate (mL/min/1.73 m^2^); CRP, C-reactive protein; AST, aspartate aminotransferase; ALT, alanine aminotransferase; TSH, thyroid-stimulating hormone; SBP, systolic blood pressure; DBP, diastolic blood pressure.

Variable Median (IQR)		Group 1 (*n* = 34)	Group 2 (*n* = 61)	Group 3 (*n* = 77)	Group 4 (*n* = 198)	*p*-Value
TC (mg/dL)		200 (169–248)	166 (142–205)	194 (167–209)	195 (168–220)	0.017
LDL (mg/dL)		119 (93–142)	94 (64.5–143)	107 (94–130)	117 (91–133)	0.071
HDL (mg/dL)		55.5 (45–68)	53.5 (46.5–71)	47 (40–55)	46 (41–53)	<0.001
TG (mg/dL)		92 (74–136)	103 (85–129)	127 (101–171)	158 (119–208)	<0.001
Non-HDL-C (mg/dL)		137.5 (102.5–180)	112 (84–153.6)	132 (116–160)	147 (122–172.5)	<0.001
Creatinine (mg/dL)		0.78 (0.66–0.88)	0.82 (0.74–0.93)	0.67 (0.58–0.78)	0.78 (0.64–0.94)	<0.001
eGFR *n*(%)	≤90	5 (15)	27 (44)	12 (16)	58 (29)	<0.001
	>90	29 (85)	34 (56)	65 (84)	132 (67)	
CRP (mg/L)		0.90 (0.40–1.40)	1.0 (0.6–1.9)	5.45 (3.3–8.6)	4.4 (2.2–8.4)	<0.001
AST (U/L)		22.5 (17.0–26.0)	23.0 (20.0–34.0)	21 (18–27)	24 (20–32)	0.029
ALT (U/L)		17.0 (15.0–24.0)	27.5 (19–40)	27 (20–38)	29 (22–47)	<0.001
TSH (µIU/mL)		1.09 (0.59–1.50)	1.1 (0.7–1.7)	1.8 (1.3–2.5)	1.5 (1.1–2.1)	<0.001
Glucose (mg/dL)		96.5 (92–102)	100 (95–111)	90 (86–99)	103 (92–123)	<0.001
Potassium (mmol/L)		4.25 (4.10–4.50)	4.3 (4.1–4.5)	4.3 (4.2–4.5)	4.2 (4.0–4.4)	0.059
SBP (mmHg)		133 (125–142)	143 (130–156)	140 (128–150)	148 (134–161)	<0.001
DBP (mmHg)		80 (77–89)	85 (77–95)	88 (80–97)	91 (80–100)	<0.001

The highest serum uric acid levels were observed in the Ob+/Ht+ group, ranging from 3.2 to 12.6 mg/dL, with a median value of 6.4 mg/dL (Figure 1).

**Table 3:** Multivariable generalized linear model assessing independent determinants of serum uric acid level. Abbreviations: BMI, body mass index; SBP, systolic blood pressure; DBP, diastolic blood pressure; TSH, thyroid-stimulating hormone; ALT, alanine aminotransferase; AST, aspartate aminotransferase; CRP, C-reactive protein; HDL, high-density lipoprotein cholesterol; TG, triglycerides; non-HDL-C, non-high-density lipoprotein cholesterol (calculated as total cholesterol minus HDL cholesterol); eGFR, estimated glomerular filtration rate (mL/min/1.73 m^2^). Model fit: AIC = 590.86.

Variable	β Coefficient	95% CI	Wald Function	*p*-Value
BMI	0.00481	0.00186–0.00775	10.2484	0.0014
SBP (mmHg)	−0.00005	−0.00179–0.00170	0.0026	0.959
DBP (mmHg)	0.00079	−0.00184–0.00341	0.3442	0.557
Glucose (mg/dL)	−0.00043	−0.00120–0.00034	1.2049	0.272
TSH (µIU/mL)	−0.01425	−0.03648–0.00799	1.5770	0.209
ALT (U/L)	0.00142	0.00004–0.00280	4.0826	0.043
AST (U/L)	−0.00021	−0.00243–0.00201	0.0342	0.853
CRP (mg/L)	−0.00006	−0.00418–0.00406	0.0008	0.977
Creatinine (mg/dL)	−0.02473	−0.23368–0.18422	0.0538	0.817
Non-HDL-C (mg/dL)	0.00020	−0.00064–0.00105	0.2195	0.639
TG (mg/dL)	−0.00009	−0.00044–0.00026	0.2530	0.615
HDL (mg/dL)	−0.00153	−0.00342–0.00036	2.5226	0.112
Age	−0.00246	−0.00463−0.00030	4.9658	0.026
eGFR ≤ 90	0.05075	0.00279–0.09871	4.3021	0.038
Sex (female)	−0.05911	−0.09593−0.02229	9.9005	0.002
Allopurinol (not receiving)	−0.07424	−0.10551−0.04298	21.6580	<0.001

## Data Availability

The data that support the findings of this study are available from the corresponding author upon reasonable request.

## Supplementary Materials

The following supporting information can be downloaded at: https://www.mdpi.com/article/10.3390/jcm15145438/s1, Figure S1. Comparison of serum uric acid levels between women and men. Data are presented as medians with interquartile ranges (25th–75th percentiles) and total ranges. The difference between groups was statistically significant (*p* < 0.001); Figure S2. Prevalence of hyperuricemia across study groups (1–4) stratified by obesity and hypertension status (Group 1: Ob−/Ht−; Group 2: Ob−/Ht+; Group 3: Ob+/Ht−; Group 4: Ob+/Ht+), presented separately for women and men; Figure S3. Linear correlation between serum uric acid level and body mass index (BMI), expressed by the correlation coefficient (r) and corresponding *p*-value; Table S1. The differences in serum uric acid level according to various characteristics; Table S2. Spearman’s correlations between serum uric acid level and a set of predictors.

## Abbreviations

The following abbreviations are used in this manuscript:

ALT	alanine aminotransferase
AST	aspartate aminotransferase
BMI	body mass index
CRP	C-reactive protein
DBP	diastolic blood pressure
eGFR	estimated glomerular filtration rate
HDL	high-density lipoprotein cholesterol
LDL	low-density lipoprotein cholesterol
non-HDL-C	non-high-density lipoprotein cholesterol
NO	nitric oxide
SBP	systolic blood pressure
TC	total cholesterol
TG	triglycerides
TSH	thyroid-stimulating hormone
WHtR	waist-to-height ratio
