# Supplementary material for: Clinical Determinants of Serum Uric Acid Levels in Patients with Obesity and Hypertension
Source: J Clin Med. 2026 Jul 11;15(14):5438. doi: 10.3390/jcm15145438 (PMC13410470; doi:10.3390/jcm15145438)
Supplement: Supplementary file 1 [file jcm-15-05438-s001.zip › Figure S1.pdf]

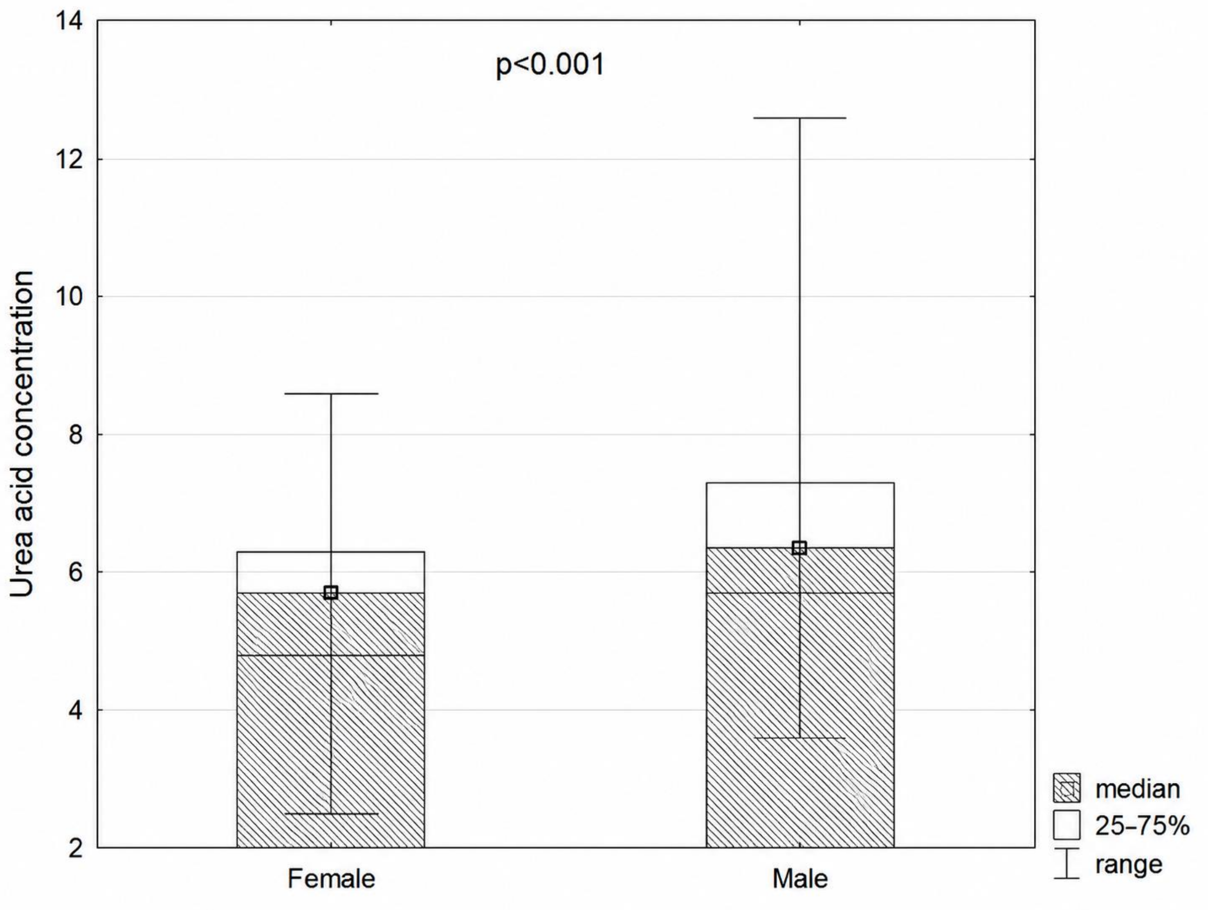

Figure S1. Comparison of serum uric acid concentrations between women and men. Data are presented as medians with interquartile ranges (25th–75th percentiles) and total ranges. The difference between groups was statistically significant ($p < 0.001$).
