# Supplementary material for: Clinical Determinants of Serum Uric Acid Levels in Patients with Obesity and Hypertension
Source: J Clin Med. 2026 Jul 11;15(14):5438. doi: 10.3390/jcm15145438 (PMC13410470; doi:10.3390/jcm15145438)
Supplement: Supplementary file 1 [file jcm-15-05438-s001.zip › Figure S2.pdf]

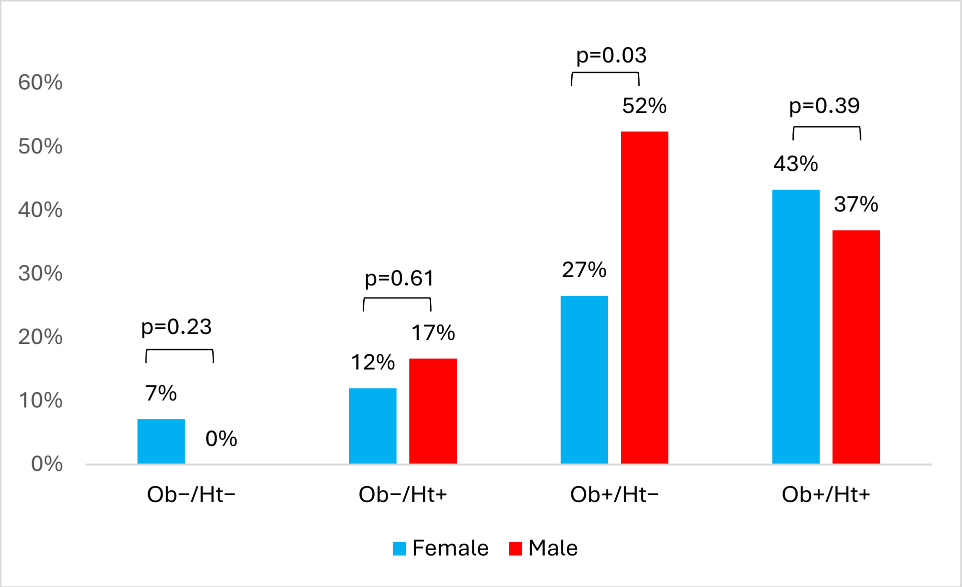

Figure S2. Prevalence of hyperuricemia across study groups (1-4) stratified by obesity and hypertension status (Group 1: Ob−/Ht−; Group 2: Ob−/Ht+; Group 3: Ob+/Ht−; Group 4: Ob+/Ht+), presented separately for women and men.
