# Supplementary material for: Clinical Determinants of Serum Uric Acid Levels in Patients with Obesity and Hypertension
Source: J Clin Med. 2026 Jul 11;15(14):5438. doi: 10.3390/jcm15145438 (PMC13410470; doi:10.3390/jcm15145438)
Supplement: Supplementary file 1 [file jcm-15-05438-s001.zip › Figure S3.pdf]

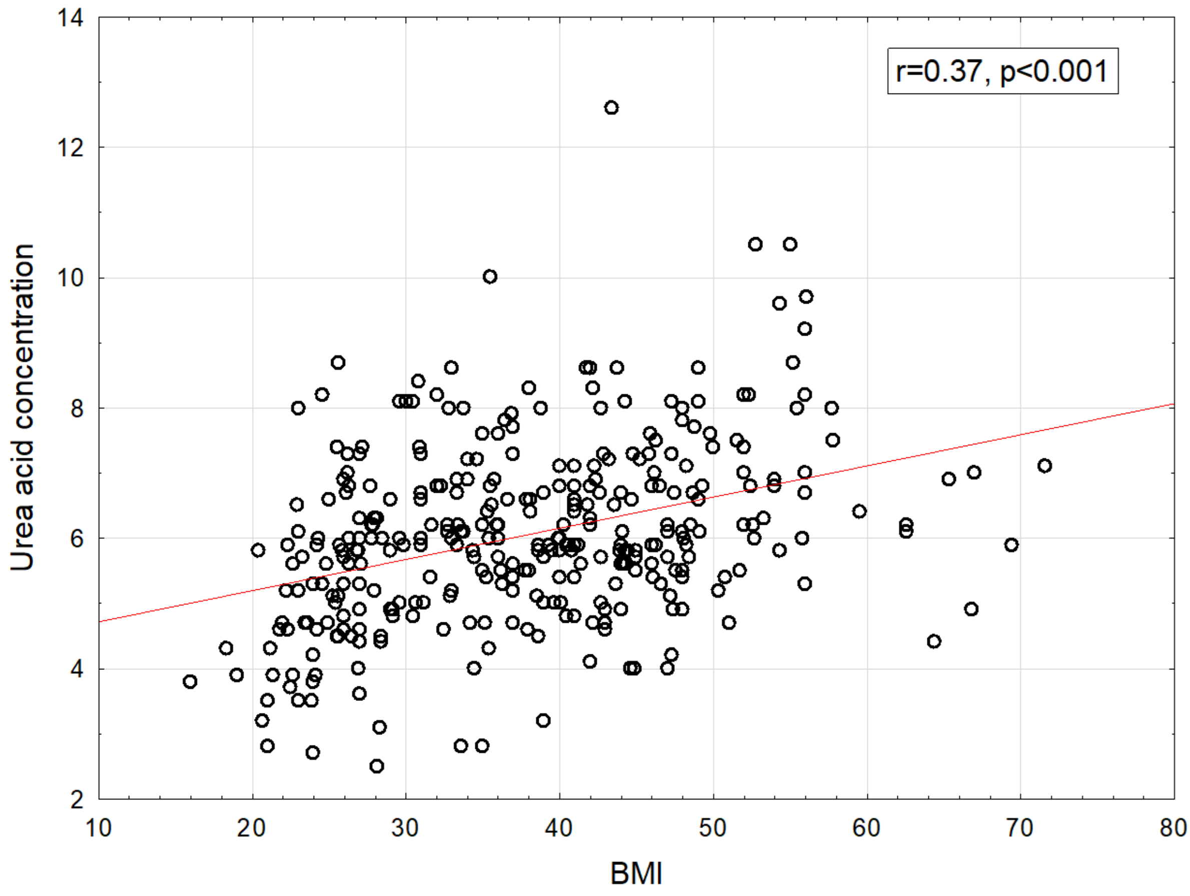

Figure S3. Linear correlation between serum uric acid concentration and body mass index (BMI), expressed by the correlation coefficient (r) and corresponding p-value.
